# Impact of circuit configuration on the mechanical performance of CPAP therapy devices: an experimental study

**DOI:** 10.3389/fmedt.2025.1508545

**Published:** 2025-02-19

**Authors:** Margherita De Luca, Andrea Formaggio, Mara Terzini, Simone Borrelli, Giovanni Putame, Francesca Moretto, Teresa Esposito, Rosanna Vaschetto, Umberto Morbiducci, Carlo Olivieri, Alberto L. Audenino

**Affiliations:** ^1^ Department of Mechanical and Aerospace Engineering, Politecnico di Torino, Turin, Italy; ^2^ PolitoBIOMed Lab, Politecnico di Torino, Turin, Italy; ^3^ Department of Translational Medicine, Università del Piemonte Orientale, Novara, Italy; ^4^ Anesthesia and Intensive Care, Azienda Ospedaliero-Universitaria Maggiore Della Carità, Novara, Italy; ^5^ Anesthesia and Intensive Care, San'tAndrea Hospital, ASL VC, Vercelli, Italy

**Keywords:** CPAP, closed configuration, ventilation circuit, experimental test bench, pressure performance, ARDS

## Abstract

**Introduction:**

CPAP therapy treats various respiratory disorders. The overall performance of therapy delivery can be affected by the adopted circuit configuration. Recently, parallel to the canonical open configuration (OC), closed configurations (CC) have been proposed with potential advantages in terms of oxygen consumption, noise, airway dryness and contamination. However, the mechanical performance of CPAP devices in CC has been marginally investigated. The aim of this study is to clarify whether CPAP therapy delivered in CC configuration retains mechanical performances equivalent to that achieved in the canonical OC stipulated by the manufacturers.

**Methods:**

OCs and CCs implemented on seven different ventilation devices, classified as flowmeter, obstructive sleep apnoea device, and mechanical ventilator, were tested at different set CPAP levels. Mask and helmet interfaces were tested, and healthy, post-surgery and ARDS respiratory conditions were simulated. The mechanical performance was compared in terms of mean static pressure (P_mean_), pressure oscillations, areas between pressure curve and P_mean_ during inspiration (A_i_) and expiration (A_e_), and the time in which the pressure curve remains above the P_mean_ along the expiration phase (T%).

**Results:**

The mechanical performances of CCs with helmet interface were comparable to canonical OCs used with mask interface. Globally, a CC supplied a reduced Pmean (on average, −1.3 cmH_2_O for the mask and −0.3 cmH_2_O for the helmet) and an increased *Δ*P, A_e_ and A_i_ (on average +0.5, +2.5, +2 times, respectively).

**Conclusion:**

The closed configuration proved its capability to effectively deliver CPAP therapy, thus making its intrinsic advantages available for future clinical use.

## 1 Introduction

Continuous positive airway pressure (CPAP) is a non-invasive ventilatory mode used to deliver a set pressure during both the patient's inhalation and exhalation phases (1). The primary objective of CPAP delivery is to avert the collapse of the upper airways and alveoli (2) and, by increasing residual functional capacity, to improve ventilatory function. Moreover, by maintaining patient's airways open and averting lung collapse, CPAP delivery enhances gas exchange efficiency. The efficacy of CPAP is intricately linked to an individual's capacity to inhale, as the therapy does not provide active assistance. CPAP is mainly delivered non-invasively to both adult and paediatric patients through different patient-ventilator interfaces (3).

CPAP therapy finds effective application in several clinical scenarios, including pulmonary oedema, respiratory distress syndrome, atelectasis, and the prevention of post-extubation respiratory failure in high-risk patients. It also serves as an effective treatment for obstructive sleep apnoea (OSA) (4). Furthermore, CPAP therapy has also demonstrated positive impacts on the cardiovascular system by reducing both cardiac preload and afterload (5, 6). During the recent SARS-CoV2 pandemic, CPAP has been widely used also to treat patients with mild or moderate ARDS non-invasively (7).

There are many commercially available devices delivering CPAP, all of them working in open configuration [including oxygen-conserving valved systems, continuous flow generators, portable demand-flow devices, mechanical ventilators (3), and OSA devices]. The canonical OC is characterized by high oxygen consumption, high noise levels for the patient, and the need for active humidification. During the SARS-CoV-2 pandemic these drawbacks, coupled with the shortage of CPAP therapy delivery devices and the risk of patient viral load dispersion in the surrounding environment, emerged prominently. To address these limitations, alternative solutions have been proposed. Among them, (i) a system allowing shared ventilation, consisting in treating multiple patients with a single ventilator while controlling air volume (8), (ii) new circuits composed of PEEP (positive end expiratory pressure) valves, antibacterial and antiviral filters that have been assembled with conventional CPAP interfaces (9–11), and (iii) solutions devised to be integrated on the ventilation circuit of CPAP machines, such as the CARL system, designed to be interposed between the CPAP device and the patient (12), or the well-known diving mask solution (13). Besides that, a CPAP closed configuration (CC) has been also proposed to address, all at once, the limitations of the standard OC delivery and the problems emerged during the SARS-CoV-2 pandemic (14). Promising numerical and *in vitro* tests investigating air pressure-flow behaviour stimulated an in-depth analysis of the performance of CPAP therapy delivered by CCs (14).The body of literature devoted to ventilation devices and their performance is mainly focused on CPAP therapy delivered with the canonical OC, employing a plethora of experimental methodologies and parameters (2, 15–20) that make comparisons challenging (Supplementary Table S1). Moreover, international standards (21) solely specify the deliverable pressure range (4–20 cmH_2_O), neglecting to define the ideal pressure performance criteria that should be warranted by the devices (such as pressure oscillations and device reactivity). Hence, systematic pressure performance results are lacking also for the canonical OC.

In this study the mechanical performance of devices capable of delivering CPAP therapy in both the OC and the CCs is evaluated through an *ad hoc* bench test with different levels of delivered therapy (i.e., CPAP pressures), interfaces, and simulated clinical conditions. To accomplish this, suitable parameters useful for pre-clinical performance comparisons are also defined and proposed. The final aim is to contribute to clarifying whether CPAP therapy delivered in CC, which can be implemented by assembling commercially available components retaining mechanical performances equivalent to that achieved in the canonical OC stipulated by the manufacturers.

## 2 Materials and methods

### 2.1 Experimental plan

**Figure 1:**
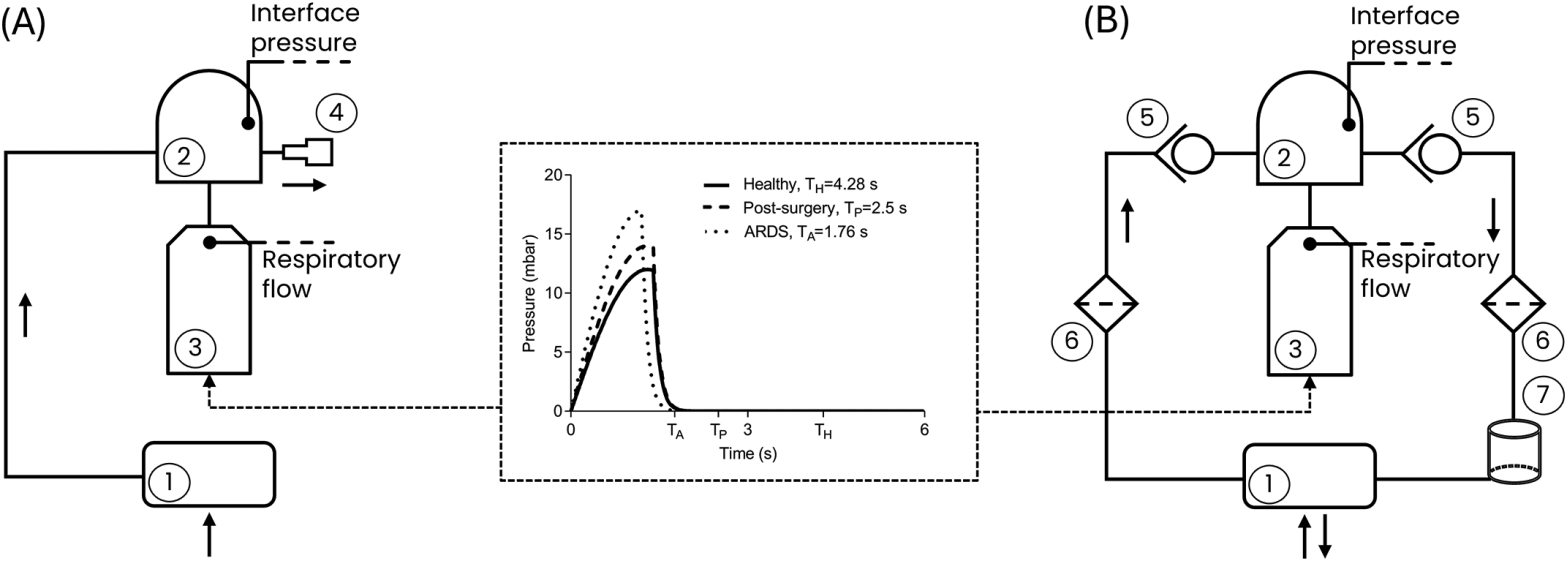
Block representation of the test bench for **(A)** the canonical open configuration and **(B)** the closed configuration. (1) CPAP device; (2) Patient interface (helmet or mask); (3) Lung simulator; (4) Expiratory valve; (5) Unidirectional valve; (6) Antiviral filter; (7) CO_2_ adsorber. Black arrows represent the flow direction. The central panel shows the respiratory effort of the three simulated clinical conditions. T_H_, T_P_ and T_A_ are the respiratory periods for the Healthy, Post-Surgery and ARDS simulated clinical conditions, respectively, determined by the respiratory rates (Table 1). The peak reached by each curve is the maximum respiratory effort (Table 1).

Seven commercial ventilation devices capable of delivering CPAP therapy were adopted: a flowmeter device [the StarVent2 from StarMed (FM)], two OSA devices [Isleep 20, Breas Medical (OSA1); AirSense 10, Resmed (OSA2)], and four mechanical ventilators configured to deliver CPAP [LUISA, Löwenstein Medical (V1); V60 Ventilator, Philips Respironics (V2); Hamilton G5, Hamilton Medical (V3); Evita V800, Draeger (V4)]. These devices are representative of those currently used in clinical settings to deliver CPAP therapy (3) and are designed by top companies manufacturing mechanical ventilators (22). The seven devices were tested using two different patient interfaces: the helmet (Castar R next, StarMed Intersurgical) and the full-face mask (MaxShield Mask, Pulmodyne, BiTrac Select). Each combination of components was tested setting three different CPAP levels, spanning the commonly used therapy range (23): 5 cmH_2_O, 7.5 cmH_2_O, and 10 cmH_2_O. The therapy delivery performances were assessed in the canonical OC as well as in the CCs. Technically, in the OC (Figure 1A) all the tested devices were directly connected to a head phantom wearing the patient interface, with the patient's breathing delivered by a lung simulator (TestChest V3, Organis Gmbh). The interface was equipped with an expiratory valve. This valve takes the form of a whisper valve when using the full-face mask interface and of a PEEP valve when using the helmet interface.

**Table 1:** Lung simulator input parameters for the simulation of three clinical conditions.

	Healthy (24–26)	Post-surgery (27–30)	ARDS (30–32)
Total respiratory compliance, Crs (ml/mbar)	100	60	40
Functional Residual Capacity, FRC (ml)	2,200	1,320	880
Respiratory rate (bpm)	14	24	34
Maximum respiratory effort (mbar/100 ms)	12	14	17

The CC (Figure 1B) adopts the same components as in the OC, with the addition of two unidirectional valves (located upstream and downstream of the patient interface, respectively, to ensure airflow unidirectionality), two antiviral filters, and a CO_2_ adsorber (specifically a soda lime canister designed to absorb the CO_2_ generated by the patient during the expiratory phase). The need for additional components for the CC is dictated by the fact that while in the OC the air exhaled by the patient is released into the surrounding environment via the expiratory valve (4), in the CC the exhaled air is routed through the unidirectional valve (5), passes through the antiviral filter (6) and the CO_2_ adsorber (7), and is then recirculated back into the circuit as purified air.

For the sake of clarity, it must be mentioned that regardless of the configuration, the tested ventilation devices retain their pressure regulation mechanisms: the FM lacks an integrated pressure sensor or feedback pressure control and therefore regulates pressure mechanically at the interface using the PEEP expiratory valve; in contrast, all other devices are equipped with pressure sensors enabling a feedback control to adjust the pressure accordingly. However, while OSA1, OSA2, and V1 measure pressure within the device's internal tubing, V2, V3, and V4 measure pressure at the patient connection port (i.e., at the patient interface).

In both OC and CCs, the air pressure generated at the patient interface (interface pressure) is recorded using a flow analyser (FlowAnalyser Pro, IMT Analytics), while the respiratory flows are recorded within the lung simulator.

Using the lung simulator, a male subject with a height of 180 cm was reproduced as a worst-case scenario. Male subjects generally have larger lung volumes and greater ventilatory demands, making them more challenging for evaluating ventilation devices mechanical performance. This subject was investigated under the healthy condition, a post-surgery hypoxemic condition, and the ARDS condition. The lung simulator settings adopted to replicate the healthy and the pathological conditions were selected based on typical clinical values reported in the literature for each condition (24–32) and are listed in Table 1 in terms of: (i) compliance of the respiratory system; (ii) functional residual capacity; (iii) respiratory rate and (iv) the maximum respiratory effort. The pressure waveforms corresponding to the set respiratory efforts prescribed to the lung simulator are presented in Figure 1.

### 2.2 Test protocol

**Figure 2:**
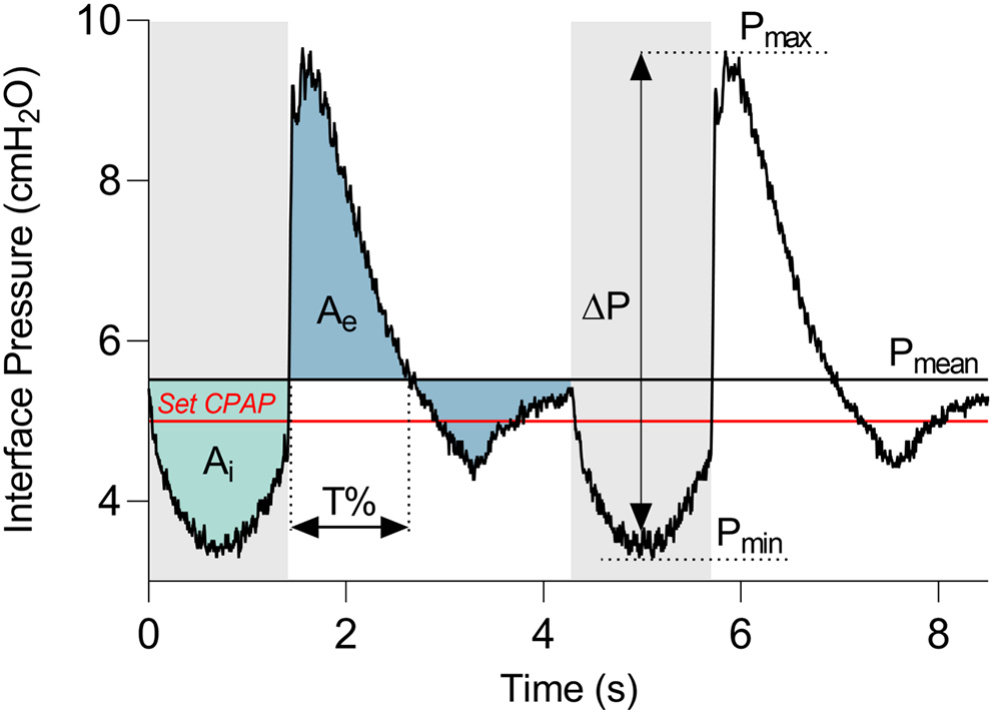
Representative curve of the pressure measured at the patient interface. Grey and white bands distinguish the expiratory and inspiratory phases. The red line indicates the set CPAP pressure. Pressure dynamic oscillation (*Δ*P) is the difference between the maximum pressure (P_max_) and the minimum pressure (P_min_). A_i_ and A_e_ are the areas between the interface pressure curve and the mean static pressure (P_mean_), during the inspiratory and the expiratory phases, respectively. T% is the time during which the curve remains above P_mean_ with respect to the entire expiration duration.

All the devices were tested according to a multistep strategy. Firstly, the device under evaluation was connected to the selected configuration (Figure 1), and the CPAP operating mode was activated on the device following the manufacturer's instructions. The CPAP level was then selected while keeping the lung simulator deactivated. At this stage, the mean static pressure level (*P*_mean_, Figure 2) reached at the patient's interface was measured. The *P*_mean_ value is an indicator of the device's capability to deliver the set therapeutic pressure at the patient interface in apnoeic condition. Subsequently, the lung simulator was activated replicating the selected clinical condition.

Once breathing stabilization was achieved, interface pressure and respiratory flow traces were recorded for a duration of 60 s. Figure 2 illustrates an example of the interface pressure curve, with the derived key parameters proposed to evaluate the pressure performance of all the devices. In detail, the amplitude of dynamic oscillations (*ΔP*) was calculated as the difference between the maximum (*P*_max_) and minimum (*P*_min_) values of the curve.

In addition, together with these commonly used pressure oscillations parameter (20), three quantities were introduced for the dynamic response assessment: the pressure deviation over time during inspiration (A_i_) and expiration (A_e_), and the persistence of the pressure curve above the *P*_mean_ during expiration (T%). The phases of inspiration and expiration (grey and white bands in Figure 2, respectively) correspond to the reversals of respiratory flow, and they were identified by detecting the time instants at which the zero-crossings of the flow curve occurred. Then, A_i_ and A_e_ were computed as the areas between the interface pressure curve and *P*_mean_ during the inspiratory and the expiratory phases, respectively. T% was quantified as the time during which the curve remains above *P*_mean_ relative to the entire expiration duration.

Contrary to the commonly used *P*_max_ or *P*_min_ parameters which are instantaneous measures, the integrated quantities A_e_ and A_i_ serve as additional descriptors of the entire breathing phases. Indeed, A_e_ and A_i_ discriminate between instantaneous high deviations from the *P*_mean_ and less pronounced but prolonged deviations. It is noteworthy that smaller expiratory and inspiratory areas, as well as a shorter T%, indicate better performance of the CPAP delivery device. In each performed test, all the values of the parameters were averaged seven breathing cycles.

### 2.3 Statistical analysis

The statistical analysis of the data was performed in MATLAB (Version R2023, MathWorks, Natick, MA, USA) environment. In detail, the normality of the statistical distribution of the quantities *Δ*P, A_i_, A_e_, T% was assessed using the Shapiro–Wilk test. Then, a multifactorial analysis of variance (Factorial ANOVA) was performed considering five independent variables (or factors): the device, the interface, the set CPAP level, the simulated clinical condition and the configuration (Table 2). The Factorial ANOVA was conducted on each dependent variable (*Δ*P, A_i_, A_e_, T%), to explore the existence of individual (i.e., main effect) or combined effects (i.e., interaction effect) of the factors on each dependent variable. The Factorial ANOVA evaluates the interaction effects exploring all possible factors combinations and statistically testing if the mean variation in the dependent variable (e.g., *Δ*P) differs for groups defined by the combination of two or more factors (e.g., device and interface). Significance levels were set to *p* < 0.05 for all tests.

**Table 2:** Experimental plan showing the tested combination of the device, simulated clinical condition, interface, and configuration factors.

	Healthy				Post-surgery				ARDS			
	Helmet		Mask		Helmet		Mask		Helmet		Mask	
	Open (OC)	Closed (CC)	Open (OC)	Closed (CC)	Open (OC)	Closed (CC)	Open (OC)	Closed (CC)	Open (OC)	Closed (CC)	Open (OC)	Closed (CC)
StarVent2 (FM)	x†				x†				x†			
Isleep 20 * (OSA1)	x	x	x†	x	x	x	x†	x	x	x	x†	x
AirSense 10 * (OSA2)	x	x	x†	x	x	x	x†	x	x	x	x†	x
LUISA* (V1)	x	x	x†	x	x	x	x†	x	x	x	x†	x
V60 ventilator* (V2)	x	x	x†	x	x	x	x†	x	x	x	x†	x
Hamilton G5 (V3)	x		x†		x		x†		x		x†	
Evita V800 (V4)			x†				x†				x†	

All tests were performed at three CPAP levels (5, 7.5, 10 cmH_2_O).

* Devices that can be tested in both open and closed configuration.

^†^Standard factors combination for the specific device as declared in the device user manual.

^x^Combinations tested.

The Partial Eta Squared $\left(\eta_{p}^{2}\right)$ was then computed as a measure of the effect size of single or combined factors. According to $\eta_{p}^{2}$ value, the impact on the dependent variable was classified as small $\left(\eta_{p}^{2}<0.01\right)$, moderate $\left(0.01\leq \eta_{p}^{2}<0.06\right)$ or large $\left(\eta_{p}^{2}\geq 0.06\right)$ (33).

## 3 Results

Table 2 comprehensively outlines all the performed tests. It can be observed, from Table 2, that some ventilation devices failed in adapting to the non-standard CC. Therefore, results are presented here solely for devices which were tested in all the possible combinations [indicated with an asterisk (*) in Table 2, first column], while all results obtained with all devices are provided in the Supplementary Tables S2–4. Overall, 180 tests were conducted.

### 3.1 Mean static pressure and pressure oscillations

**Figure 3:**
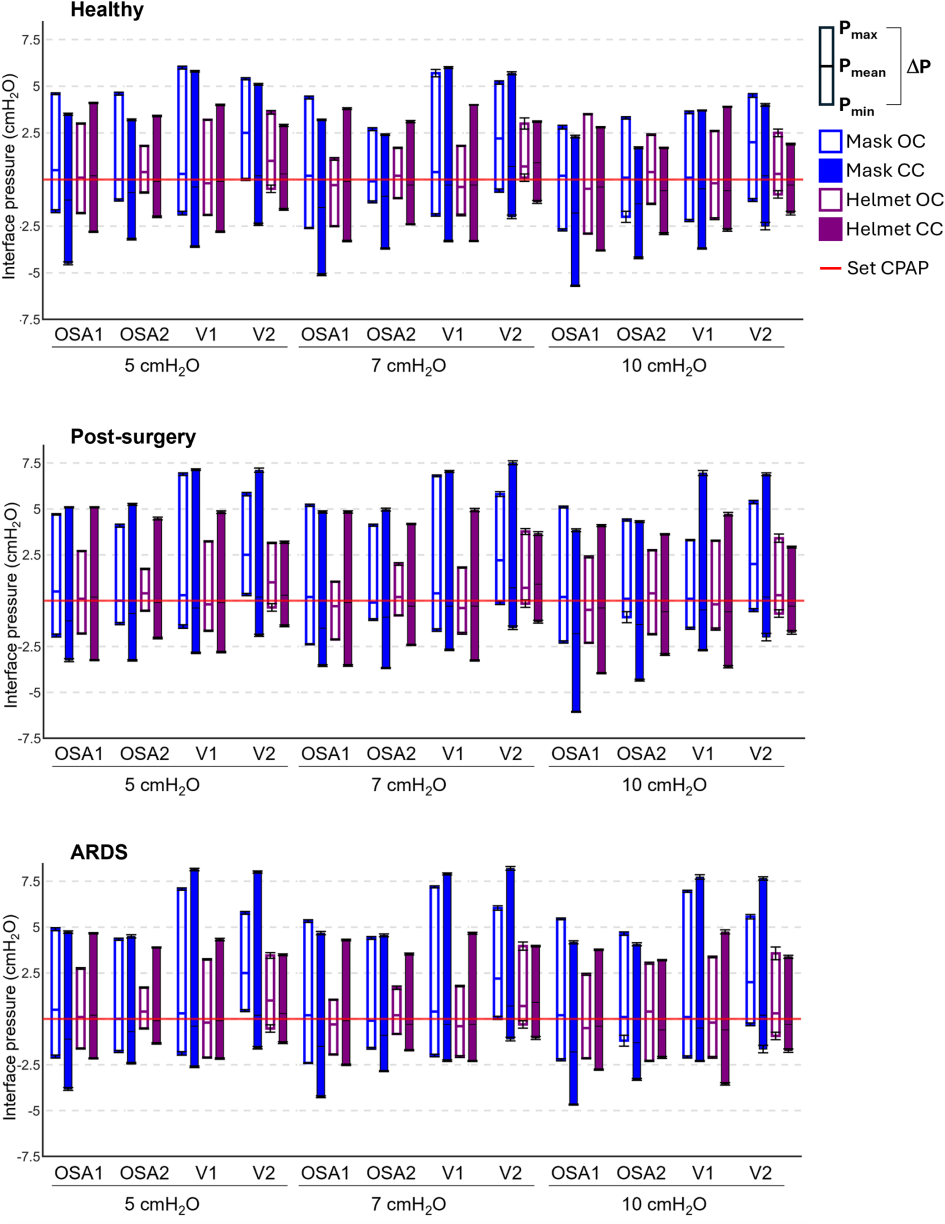
Pressure performances of the four devices tested in all the possible combinations. Each box plot represents the averaged pressure parameters computed in each tested combination: lower extremity is P_min_, upper extremity is P_max_, the intermediate line is P_mean_, the box height is *Δ*P. The red line is the set CPAP. Mask OC, mask interface with the open configuration; Mask CC, mask interface with the closed configuration; Helmet OC, helmet interface with the open configuration; Helmet CC, helmet interface with the closed configuration. Numerical values are also reported in the Supplementary Tables S2–4.

Figure 3 provides the P_mean_ values and the *Δ*P values for each performed test at the three set CPAP levels investigated.

Notwithstanding the favorable apnoeic condition, in all the devices P_mean_ values deviate from the set CPAP level regardless of the configuration and the set CPAP level (Figure 3). Closing the ventilation circuit globally leads to a P_mean_ reduction, with major impact when the mask is used (−1.3 cmH_2_O on average for the mask against −0.3 cmH_2_O on average for the helmet). This reduction in P_mean_ values is scarcely affected by the set CPAP level.

The results highlight that closing the ventilation circuit invariably leads to an increase in *Δ*P (an indicator of the devices reactivity) regardless of the simulated clinical condition under test, of the device, and of the set CPAP pressure level, with an average rise of 50% compared to the corresponding OC. In some cases, increments up to three times emerge for *Δ*P values.

**Figure 4:**
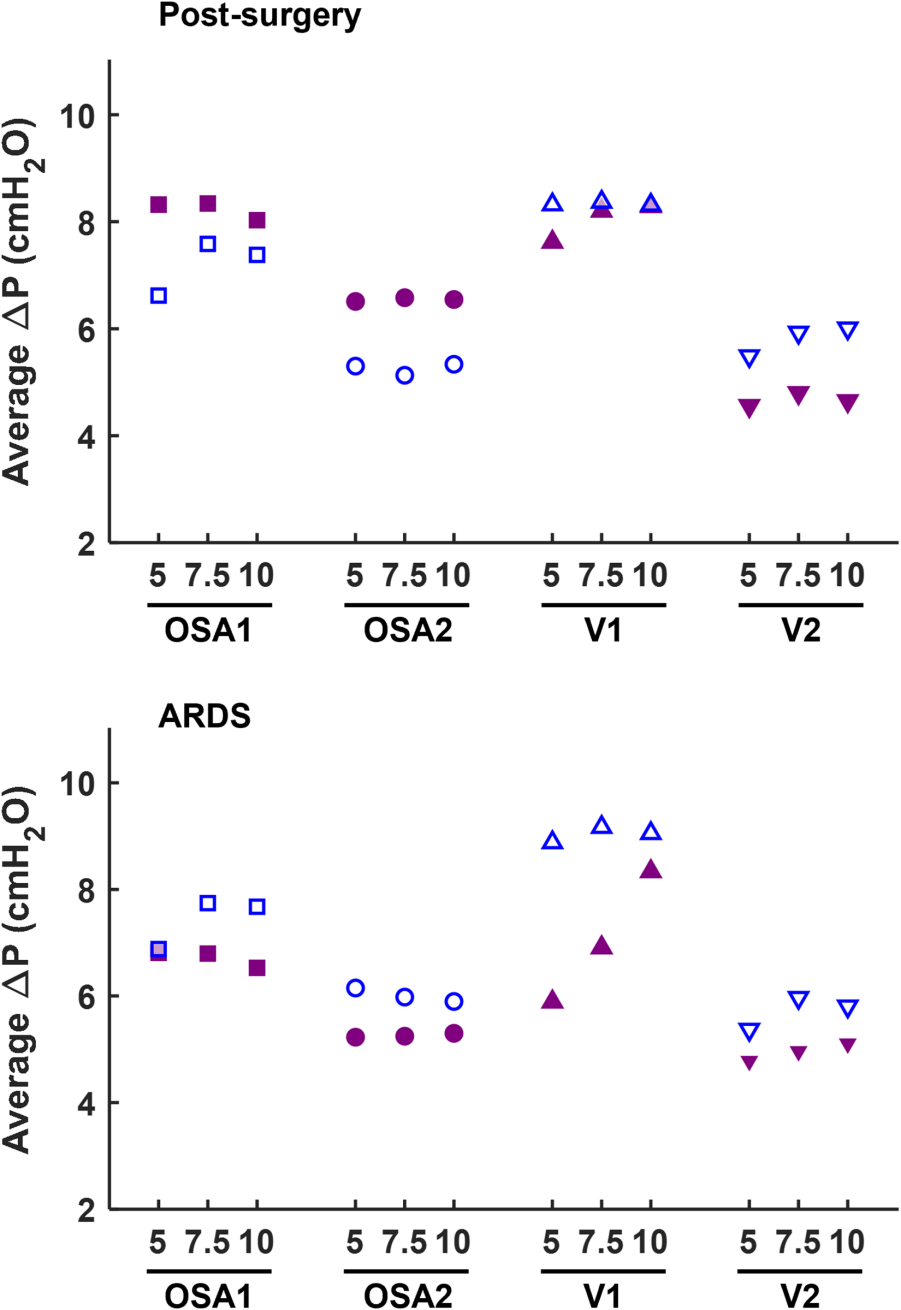
Comparison of *Δ*P measured at the patient interface between the canonical open configuration with the mask (empty dots) and the new closed configuration with the helmet interface (filled dots).

Conversely, increasing the patient interface volume (i.e., replacing the mask with the helmet) always reduces *Δ*P values, with decrements ranging from 10% to 64%. Therefore, the increase in *Δ*P induced by the ventilation circuit closure can be compensated for by the patient's interface. In this regard, Figure 4 highlights that adopting a larger interface confers to the CC a performance which is similar to (and often with smaller pressure oscillations) the canonical OC with the mask interface.

Focusing on the impact of the administered CPAP level, its influence on *Δ*P varies depending on the interface, configuration, or simulated clinical condition. Although a direct correlation between *Δ*P and the set CPAP levels does not robustly emerge, insights can be gained by examining the deviations in *Δ*P across the three CPAP levels, computed as the largest offset between the three *Δ*P values and their average. Employing the OC, *Δ*P values deviation is always above 10% in the healthy simulated condition, regardless of the interface adopted. However, in post-surgery and ARDS simulated conditions *Δ*P deviation never exceeds 8% with the mask, but always surpass 8% (max. 45% for post-surgery and 59% for ARDS) with the helmet almost in all combinations (7 out of 8). Conversely, the CC presents lower *Δ*P deviations across the three set CPAP pressure levels investigated, especially for OSA devices. Indeed, OSA1 and OSA2 exhibit *Δ*P deviations below 4% in pathological simulated conditions, while larger but <7% *Δ*P deviations are obtained using V2 and V1, the latter only for the post-surgery simulated condition. As for V1, *Δ*P deviations nearly reaching 20% emerge for the ARDS simulated condition.

Finally, comparing the different simulated clinical conditions, pathological ones exhibit higher *Δ*P values. This is primarily due to an increase in the expiratory peak (P_max_), while the inspiratory peak (P_min_) remains relatively unchanged. The observed higher P_max_ values are ascribable to the pathologic simulated conditions' higher respiratory rate.

Elevated P_max_ values are particularly noticeable when using a mask interface, as the combination of the smaller interface volume and of the higher respiratory rate makes it harder for the controller of the devices the minimization of pressure oscillations. However, the use of the helmet interface proves effective in mitigating this issue. Furthermore, in the OC, as the severity of the pathology worsens (i.e., moving from post-surgery to ARDS), there is a general increase in *Δ*P values. Notably, in the ARDS simulated condition *Δ*P values are higher (up to +20%) compared to the post-surgery one. Interestingly, when the ventilation circuit is closed, in most combinations, larger *Δ*P oscillations (up to +20%) are observed for the post-surgery condition, compared to the ARDS one.

### 3.2 Expiratory area vs. Inspiratory area

**Figure 5:**
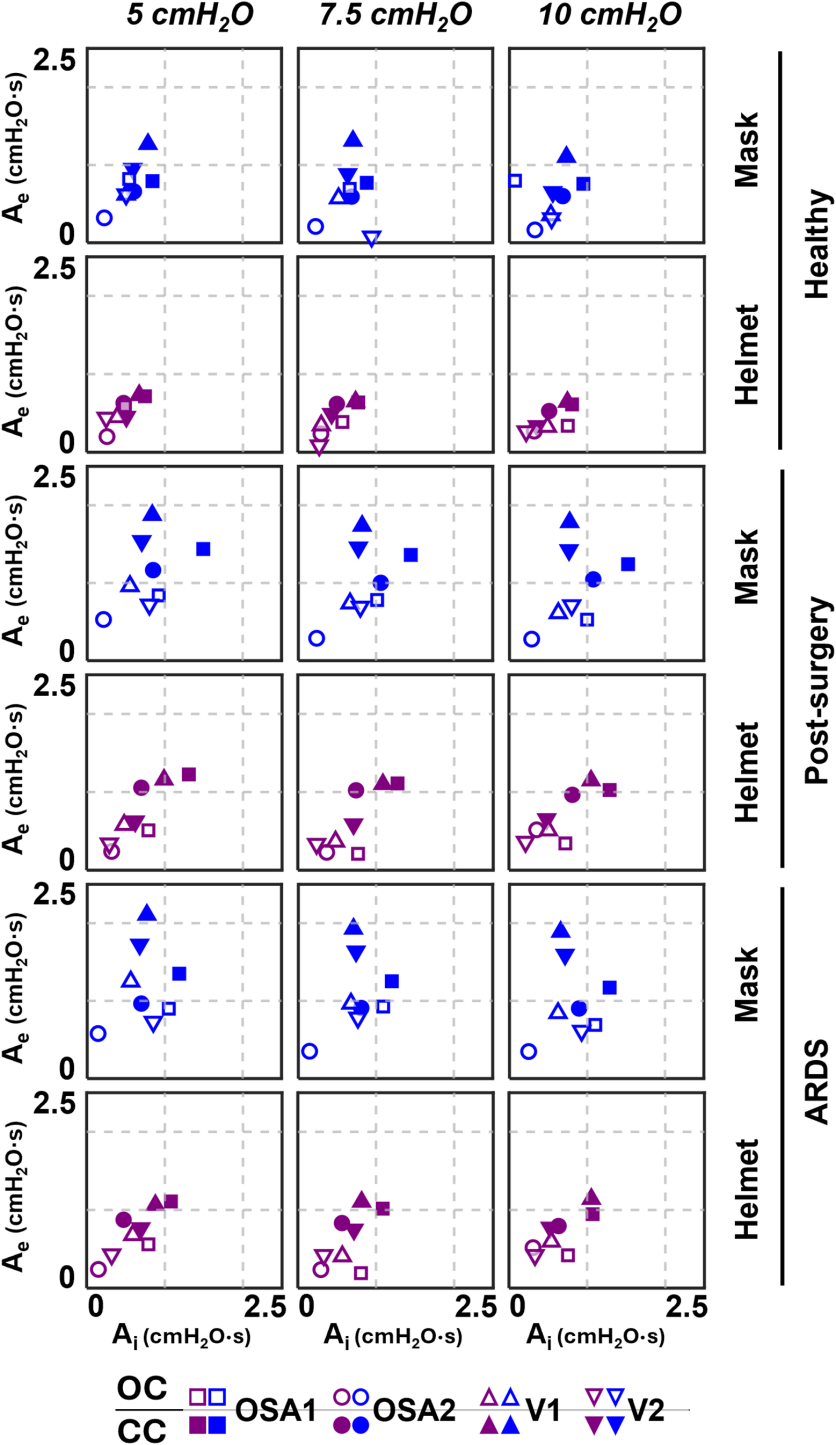
A_e_-A_i_ results of the four devices.

The comparison expiratory area A_e_ vs. inspiratory area A_i_ is presented in Figure 5. Like *Δ*P data, closing the ventilation circuit leads to an overall increase of the areas, with major impact on A_e_. Indeed, on average, a 2.5 times increment is recorded on A_e_ with peaks reaching 13 times, while a 2 times increment is recorded on A_i_ with peaks reaching 11 times. This is explained by the positive correlation between the *Δ*P and the expiratory area (*R*^2^ > 0.76). Moreover, both A_i_ and A_e_ are not affected by the administered CPAP set level.

Overall, A_i_ and A_e_ are larger in simulated pathological conditions, with a major increase for A_e_ (+38% on average) then for A_i_ (+27% on average) with respect to the simulated healthy condition.

### 3.3 Expiratory area vs. T%

**Figure 6:**
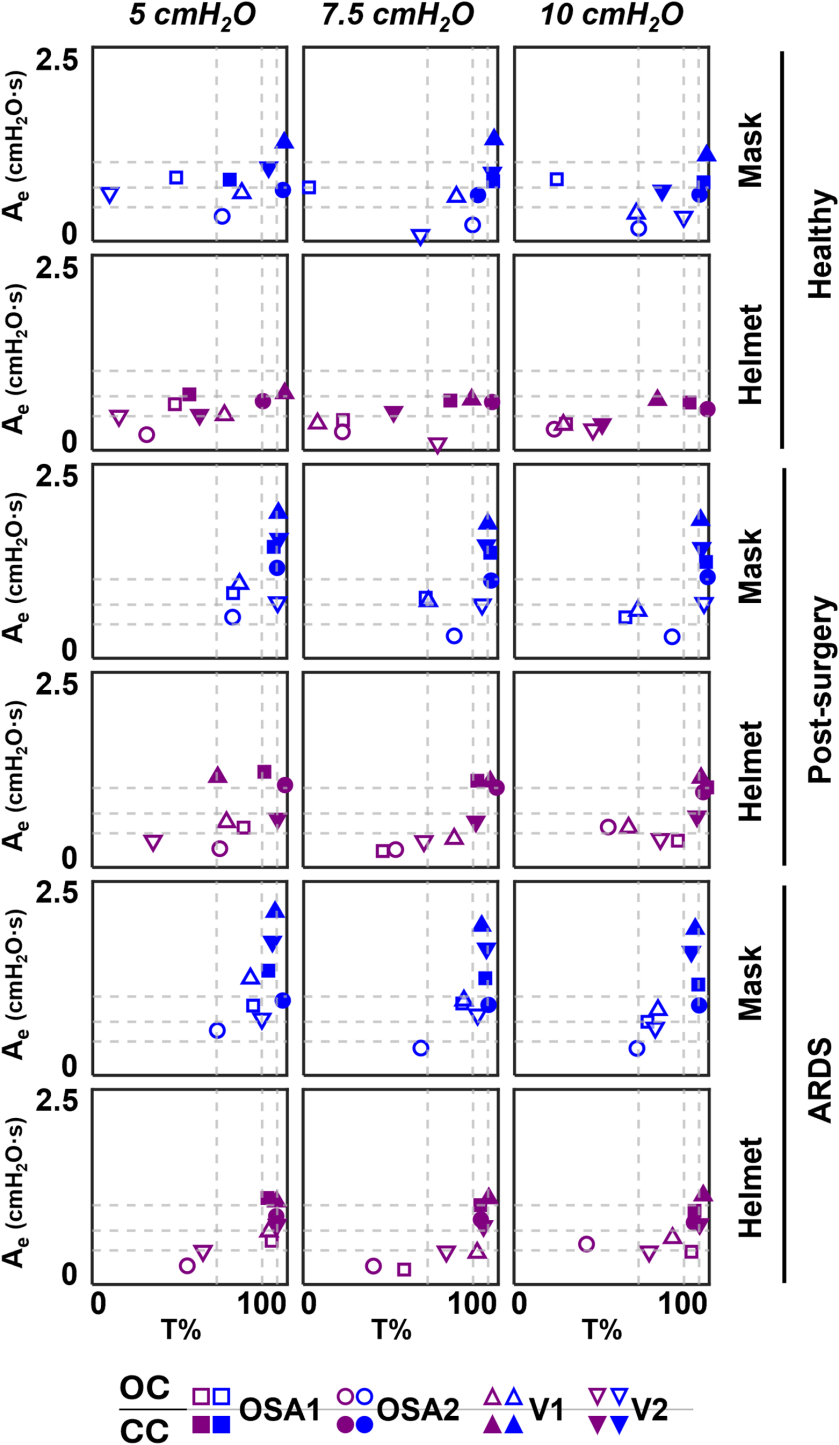
A_e_-T% results of the four devices. Dotted lines correspond to the 25th, 50th and 75th percentiles computed separately on both Ae (horizontal lines) and on T% (vertical lines) parameters.

Further considerations can be drawn from the A_e_ vs. T% relationships reported in Figure 6, where it emerges that moving from the top-right to the bottom-left corner, the combinations exhibit increasingly responsive behaviour.

For our purposes, this responsiveness is defined as the device's ability to minimize pressure oscillations while working within that specific combination of interface, set CPAP level, simulated clinical condition, and configuration.

Focusing on the configurations, OC is mainly located below 50th percentile of A_e_, while CC is mainly located above, implying worse responsiveness. The introduction of the helmet interface shifts both configurations towards lower A_e_. Moreover, in OC, it also reduces the time during which the curve remains above P_mean_ with respect to the entire expiration duration (i.e., T%); this doesn't occur in CC for pathological simulated conditions.

### 3.4 Statistical analysis

Finally, the multifactorial analysis of variance highlighted that all the investigated factors have a significant impact on *Δ*P, A_i_, A_e_, T%. Interface, device, and configuration variables showed a strong effect, as indicated by very low *p*-values (≪0.05), while the set CPAP level has a less pronounced effect. Specifically, no significant effects were observed in the following: (1) for the A_i_ parameter, in the interactions between set CPAP level and configuration (*p* = 0.47), set CPAP level and simulated clinical condition (*p* = 0.10), and set CPAP level and interface (*p* = 0.096); (2) for the A_e_ parameter, in the interaction between set CPAP level and simulated clinical condition (*p* = 0.26); and (3) for the T% parameter, with the set CPAP level factor alone (*p* = 0.16). The low variability observed in all the measured quantities confirms that the CC is stable and robust in delivering CPAP. Detailed factorial ANOVA results are provided in the Supplementary Tables S5–8.

The Partial Eta Squared analysis allowed to rank the factors according to their effect size on each dependent variable. For *Δ*P, the interface showed the largest effect (0.27), while the configuration had a smaller but still notable impact (0.19). For A_i_ and A_e_, the configuration was the most dominant factor (0.14 and 0.28, respectively), while the interface showed a moderate effect (0.06 and 0.15, respectively). The configuration and the simulated clinical condition showed relevant effects on T% (0.19 and 0.12, respectively). Finally, the set CPAP level had minimal impact on all dependent variables, with $\eta_{p}^{2}$ consistently below 0.01.

## 4 Discussion

The CC (14) could introduce important advantages in CPAP delivery mitigating shortcomings of the current technologies. Indeed, continuous flow generators (gold-standard technology for CPAP delivery), OSA homecare devices and mechanical ventilators require high oxygen flow and active gas humidification due to their functioning in open configuration. Continuous flow generators are also responsible for noise generation due to their working principle based on high flows. The CC considerably reduces oxygen consumption, noise and does not require any humidification, being it provided by the patient itself. To transfer this concept to the bedside, and thus leveraging the advantages described above, it is necessary to assess whether and how this new closed configuration performs comparably to the canonical open configuration. Therefore, a systematic investigation of pressure performance with commercial devices capable of delivering CPAP was undertaken.

To this purpose, five factors were combined: device*,* interface*,* set CPAP level, simulated clinical condition and configuration. The Factorial ANOVA conducted highlights the significant impact of device, interface, simulated clinical condition and configuration factors on *Δ*P, A_i_, A_e_, and T%. This underscores the crucial role these factors play in influencing the mechanical performance of CPAP systems. In contrast, the set CPAP level demonstrated a less pronounced effect compared to the other factors. Moreover, the low variability observed across all measured quantities reinforces the stability and reliability of the CC in delivering CPAP therapy.

Not all devices allow ventilation circuit closure, either due to incompatibility with the device functioning mechanisms (FM) or device inner software limitations, which led to therapy delivery stoppage and alarm activation (V3 and V4). The devices tested in both OC and CC were analysed in all factor combinations, although the canonical configuration declared in all the devices' user manual is an open configuration with a mask interface.

Closing the ventilation circuit with the helmet resulted in pressure performances similar and often superior to the canonical configuration as clearly illustrated in Figure 4. However, keeping the same interface, closing the ventilation circuit consistently led to a decrease in performance in terms of *Δ*P, A_e_ and T% increase. In the CC, exhaled air is recirculated and filtered through the introduction of additional components such as unidirectional valves, antivirals filters and CO_2_ adsorbers, which inherently increase circuit resistance. This increased resistance, coupled with the absence of intentional leaks into the environment, accounts for the observed increase in pressure oscillations. The adoption of a larger interface, success in balancing the *Δ*P and A_e_ increase. As expected, larger volumes mitigate pressure oscillations by amplifying the air compressibility effect. However, this strategy scarcely affects T%, suggesting that T% is more closely linked to the device inner control rather than the configuration, and therefore intervention on the former is needed to correct the poorer performance. Observing Figure 6, it is clear that even canonical configurations do not fall into the lower left corner of the A_e_-T% plane, region linked to the best obtainable performance (i.e., low A_e_ and low T%). This suggests that designing a specific device tailored for use in the new closed configuration would be beneficial: a dedicated inner control would have the capability to reduce T% below the 50th percentile, aligning with the performance of most of the canonical configurations, in pathological simulated conditions. Conversely, A_i_, which is associated to a temporary reduction of the delivered CPAP level due to patient's inspiration, is less influenced by the ventilation circuit closure (Figure 5).

It is important to note that comparisons did not include the flowmeter, only evaluated in open loop configuration with the helmet. Since it operates in an advantageous configuration in relation to pressure oscillations, the flowmeter performance was unsurprisingly the most optimal (Supplementary Tables S2–4). However, it amplifies the shortcomings of the OC, exhibiting the highest levels of oxygen consumption, noise, and airway dryness. While the CC with the helmet does not achieve the same performance levels as the flowmeter, its introduction enables the mitigation of the shortcomings of open ventilation circuits, maintaining performance comparable to other solutions frequently adopted in clinical practice, such as ventilators in OC with the mask. Given that, future research will be necessary to confirm whether this CC provides the anticipated theoretical benefits, such as reducing viral load contamination, decreasing daily oxygen consumption, minimizing device noise, and eliminating the need for active humidification to enhance long-term adherence to the therapy.

It's noteworthy that this study questions the ventilation devices mechanical performance in delivering CPAP therapy, while does not claim to propose an ideal patient-specific pressure selection criterion, as this lies beyond the scope of the present work. Due to the diverse population requiring mechanical ventilation, achieving an ideal criterion that suits all patients is, indeed, unlikely. The optimal pressure level should, simultaneously, ensure adequate gas exchange, maintain lung openness to prevent phasic airway collapse, avoid overdistension of alveoli, and not adversely affect hemodynamic (34–36). These goals may be attainable at different pressure levels, and selecting the appropriate one always involves a compromise among these objectives. Nevertheless, once the optimal pressure level is determined, the ventilation device must ensure the correct delivery of therapy. Results highlighted that, with currently employed devices, the delivery of the therapy not always adhere with the CPAP level set by clinicians. For instance, deviations from the set CPAP level up to 2.5 cmH_2_O in static conditions occurred also during canonical use of the studied devices (OC with the mask in Figure 3). The impact on the treatment outcomes of the deviations here reported is hard to quantify. Nevertheless, the variability encountered between the tested devices implies non-uniformities in therapy delivery among clinical structures, also undermining the adoption of common guidelines for CPAP therapy.

There are some limitations that could weaken the findings of this study. Lung simulators (i) do not mimic the large intrasubject breathing variability, and (ii) are set to deliver performances based on quantities averaged over specific populations (healthy, post-surgery or ARDS simulated conditions). Therefore, the lung simulator's settings are not able to capture the full range of responses to mechanical ventilation observed in real patients with complex comorbidities or varying degrees of disease severity. Moreover, the lung simulator sustains a maximum P_mean_ value up to 10 cmH_2_O, while in clinical practice therapies at CPAP levels up to 15 cmH_2_O can be also delivered. Based on this study results, a higher CPAP level would majorly influence the OC rather than the CC one. Despite these limitations, the here presented results offer valuable insights into the mechanical performance of the CPAP device in a controlled environment. However, further studies involving human subjects will be necessary to assess the clinical relevance of these findings to real-world settings.

## 5 Conclusion

In conclusion CPAP therapy delivered with the new closed configuration using commercial devices together with a helmet interface, retains the pressure performances equivalent to those reached in the canonical open configuration, regardless the CPAP set level or the clinical condition simulated. The closed configuration proves valuable for its further development through a device tailored for working in closed configuration, thus making its intrinsic advantages available for future clinical use: further studies are needed to test safety and efficacy.

## Data Availability

The raw data supporting the conclusions of this article will be made available by the authors, without undue reservation.
