# Supplementary material for: Impact of circuit configuration on the mechanical performance of CPAP therapy devices: an experimental study
Source: Front Med Technol. 2025 Feb 19;7:1508545. doi: 10.3389/fmedt.2025.1508545 (PMC11879946; doi:10.3389/fmedt.2025.1508545)
Supplement: Supplementary file 1 [file Datasheet1.pdf]

Supplementary Materials

for

Impact of circuit configuration on the mechanical performance of CPAP therapy devices: an experimental study

Margherita De Luca[a, b]*; Andrea Formaggio[a, b]; Mara Terzini[a, b]; Simone Borrelli[a,b] ; Giovanni Putame[a,b] ; Francesca Moretto[c,d] ; Teresa Esposito[c,d] ; Rosanna Vaschetto[c,d] ; Umberto Morbiducci[a,b] ; Carlo Olivieri[e] ; Alberto L. Audenino[a,b]

[a] Department of Mechanical and Aerospace Engineering, Politecnico di Torino, Turin, Italy

[b] Polito[BIO]Med Lab, Politecnico di Torino, Turin, Italy

[c] Department of Translational Medicine, Università del Piemonte Orientale, Novara, Italy

[d] Anesthesia and Intensive Care, Azienda Ospedaliero-Universitaria Maggiore Della Carità, Novara, Italy.

[e] Sant'Andrea Hospital, ASL VC, Vercelli, Italy

* Corresponding author: margherita.deluca@polito.it

Supplementary tables list

Table S1: Literature review of experimental evaluations of ventilation devices delivering CPAP therapy, with particular reference on pressure performance.

Table S2: ΔP, A_i, A_e and T% results (mean ± standard deviation) for all performed tests at 5 cmH_2O.

Table S3: ΔP, A_i, A_e and T% results (mean ± standard deviation) for all performed tests at 7.5 cmH_2O.

Table S4: ΔP, A_i, A_e and T% results (mean ± standard deviation) for all performed tests at 10 cmH_2O.

Table S5: Factorial ANOVA table for the ΔP parameter.

Table S6: Factorial ANOVA table for the A_i parameter.

Table S7: Factorial ANOVA table for the A_e parameter.

Table S8: Factorial ANOVA table for the T% parameter.

Table S1: Literature review of experimental evaluations of ventilation devices delivering CPAP therapy, with particular reference on pressure performance.

References	Tested Device	Tested Pressures (cmH_2O)	Interface	Subject	Measured parameters	Aim
Abdenbi et al. [15]	OSA	4	Starling resistor		Residual hypopnoea indices; Reaction time; Pmax	Ability to detect respiratory events and adjust pressure accordingly
Lofaso et al. [18]	OSA	8, 12			P max; Flattening index; Tidal volume	Ability to detect various shapes of inspiratory flow contour and the and the corresponding response of the device
Fauroux et al. [17]	Ventilator			Paediatric profiles	Airway pressure; Tidal volume; Flow	Performance characteristics of the ventilators in children
Glover GW et al. [19]	Flowmeter (Whisperflow)	5, 10, 15	Mask		Flow; CPAP load; Pmean; Pmin	Technical performance of the Whisperflow and the CPAP valves
Louis et al. [20]	OSA	15	Nasal pillow	Overweight patient	ΔP*; PEEP; [illegible]T**; Pinsp mean; Pexp mean; WOB exp; WOB insp	Measurement of the additional WOB imposed by the devices
Chen Z-L et al. [16]	OSA	8	Nasal mask		Pmax; Pmin; ΔT1**; Kc***; ΔT2****	Pressure stability comparison between experimental and commercial devices
Brusasco et al.[2]	Flowmeter	5, 10, 15, 20	Orofacial mask Helmet		Flow; Pressure; FiO_2; Oxygen consumption	Intrinsic capabilities of devices in prehospital and emergency settings

* from PEEP to the minimal value during inspiration.
** time interval from inspiration onset to the minimal airway pressure value.
*** the ration of pressure drop during inspiration to time interval ΔT1.
**** time interval during which airway pressure is greater than the pre-set pressure.

Table S2: ΔP, A_i, A_e and T% results (mean ± standard deviation) for all performed tests at 5 cmH_2O

		Healthy				Post-surgery				ARDS			
		Mask		Helmet		Mask		Helmet		Mask		Helmet	
		Open (OC)	Closed (CC)	Open (OC)	Closed (CC)	Open (OC)	Closed (CC)	Open (OC)	Closed (CC)	Open (OC)	Closed (CC)	Open (OC)	Closed (CC)
**StarVent2 (FM)**	**ΔP**			3.00 ± 0.40				3.96 ± 0.03				2.91 ± 0.05	
	**A_i**			0.317 ± 0.056				0.394 ± 0.004				0.425 ± 0.051	
	**A_e**			0.327 ± 0.077				0.440 ± 0.004				0.439 ± 0.051	
	**T%**			54.9 ± 11.2				97.8 ± 0.5				83.8 ± 32.2	
**Isleep 20 (OSA1)**	**ΔP**	6.41 ± 0.09	8.03 ± 0.08	4.77 ± 0.04	6.92 ± 0.05	6.63 ± 0.06	9.94 ± 0.07	4.49 ± 0.04	8.33 ± 0.03	6.94 ± 0.08	8.55 ± 0.08	4.36 ± 0.05	6.82 ± 0.04
	**A_i**	0.540 ± 0.004	0.841 ± 0.006	0.591 ± 0.003	0.745 ± 0.003	0.921 ± 0.006	1.491 ± 0.006	0.786 ± 0.004	1.310 ± 0.004	1.061 ± 0.036	1.181 ± 0.008	0.780 ± 0.006	1.078 ± 0.005
	**A_e**	0.816 ± 0.006	0.789 ± 0.008	0.490 ± 0.004	0.717 ± 0.009	0.840 ± 0.005	1.442 ± 0.008	0.515 ± 0.005	1.223 ± 0.006	0.896 ± 0.046	1.353 ± 0.005	0.563 ± 0.005	1.116 ± 0.030
	**T%**	43.2 ± 0.5	69.4 ± 3.8	42.2 ± 0.6	49.8 ± 1.3	72.0 ± 0.7	93.0 ± 0.6	77.7 ± 1.1	88.2 ± 0.7	75.3 ± 23.5	90 ± 0.6	92.1 ± 0.6	89.9 ± 0.9
**AirSense 10 (OSA2)**	**ΔP**	5.68 ± 0.07	6.35 ± 0.07	2.50 ± 0.05	5.36 ± 0.05	5.34 ± 0.09	8.50 ± 0.07	2.28 ± 0.04	6.51 ± 0.05	6.12 ± 0.09	6.92 ± 0.10	2.22 ± 0.04	5.23 ± 0.04
	**A_i**	0.222 ± 0.006	0.601 ± 0.004	0.116 ± 0.007	0.471 ± 0.005	0.209 ± 0.005	0.908 ± 0.003	0.327 ± 0.005	0.743 ± 0.016	0.137 ± 0.005	0.702 ± 0.040	0.148 ± 0.006	0.473 ± 0.005
	**A_e**	0.315 ± 0.007	0.656 ± 0.006	0.422 ± 0.012	0.632 ± 0.005	0.531 ± 0.005	1.108 ± 0.004	0.238 ± 0.006	1.066 ± 0.011	0.579 ± 0.006	0.968 ± 0.006	0.238 ± 0.004	0.877 ± 0.005
	**T%**	67.7 ± 2.8	97.4 ± 1.3	19.5 ± 0.4	89.0 ± 2.6	72.1 ± 0.5	94.8 ± 0.5	63.8 ± 2.5	99.1 ± 0.3	64.4 ±1.2	97.5 ± 0.7	48.3 ± 0.5	94.9 ± 0.5
**LUISA (V1)**	**ΔP**	7.83 ± 0.06	9.27 ± 0.05	5.09 ± 0.04	6.86 ± 0.05	8.30 ± 0.07	9.99 ± 0.04	4.87 ± 0.05	7.62 ± 0.08	9.0 ± 0.08	10.75 ± 0.08	5.35 ± 0.03	6.34 ± 0.04
	**A_i**	0.488 ± 0.019	0.782 ± 0.003	0.387 ± 0.003	0.672 ± 0.003	0.559 ± 0.010	0.830 ± 0.006	0.481 ± 0.006	1.100 ± 0.117	0.591 ± 0.030	0.761 ± 0.011	0.583 ± 0.009	0.975 ± 0.030
	**A_e**	0.622 ± 0.018	1.263 ± 0.015	0.447 ± 0.012	0.723 ± 0.011	0.952 ± 0.008	1.881 ± 0.007	0.582 ± 0.006	1.046 ± 0.113	1.245 ± 0.007	2.115 ± 0.009	0.682 ± 0.008	1.022 ± 0.020
	**T%**	83.4 ± 8.8	97.6 ± 1.2	67.8 ± 1.1	98.7 ± 1.0	74.7 ± 0.6	95.3 ± 0.4	68.7 ± 0.7	21.2 ± 39.0	80.8 ± 0.6	93.8 ± 0.7	90.1 ± 0.7	87.4 ± 2.8
**V60 ventilator (V2)**	**ΔP**	5.45 ± 0.08	7.51 ± 0.25	4.12 ± 0.23	4.48 ± 0.09	5.46 ± 0.09	9.00 ± 0.13	3.51 ± 0.05	4.56 ± 0.06	5.33 ± 0.09	9.36 ± 0.08	3.98 ± 0.15	4.79 ± 0.06
	**A_i**	0.500 ± 0.003	0.618 ± 0.098	0.228 ± 0.040	0.511 ± 0.009	0.798 ± 0.008	0.704 ± 0.010	0.292 ± 0.017	0.629 ± 0.017	0.846 ± 0.007	0.720 ± 0.010	0.341 ± 0.029	0.709 ± 0.021
	**A_e**	0.623 ± 0.003	0.926 ± 0.088	0.437 ± 0.019	0.462 ± 0.015	0.715 ± 0.008	1.556 ± 0.016	0.340 ± 0.007	0.625 ± 0.008	0.728 ± 0.007	1.707 ± 0.010	0.421 ± 0.018	0.771 ± 0.016
	**T%**	9.9 ± 0.5	93.2 ± 13.5	13.77 ± 2.5	45.8 ± 3.3	95.0 ± 1.5	96.1 ± 0.4	30.8 ± 0.6	95.2 ± 0.3	86.7 ± 0.9	89.6 ± 16.4	56.6 ± 4.3	94.5 ± 0.7
**Hamilton G5 (V3)**	**ΔP**	9.08 ± 0.40		5.60 ± 0.14		9.12 ± 0.20		4.44 ± 0.24		10.34 ± 0.87		4.827 ± 0.66	
	**A_i**	1.093 ± 0.037		0.768 ± 0.009		0.264 ± 0.060		0.484 ± 0.025		0.323 ± 0.022		0.420 ± 0.098	
	**A_e**	0.143 ± 0.006		0.295 ± 0.014		1.437 ± 0.009		0.795 ± 0.100		1.712 ± 0.0.069		1.007 ± 0.121	
	**T%**	48.3 ± 4.02		59.9 ± 1.3		95.8 ± 1.8		99.6 ± 1.1		92 ± 1.2		99.9 ± 0.2	
**Evita V800 (V4)**	**ΔP**	9.21 ± 0.22				9.74 ± 0.19				11.96 ± 0.42			
	**A_i**	0.170 ± 0.021				0.321 ± 0.059				0.496 ± 0.164			
	**A_e**	0.421 ± 0.027				0.589 ± 0.010				0.761 ± 0.171			
	**T%**	92.3 ± 3.1				84.0 ± 3.4				92.5 ± 7.9			

Table S3: ΔP, A_i, A_e and T% results (mean ± standard deviation) for all performed tests at 7.5 cmH_2O

		Healthy				Post-surgery				ARDS			
		Mask		Helmet		Mask		Helmet		Mask		Helmet	
		Open (OC)	Closed (CC)	Open (OC)	Closed (CC)	Open (OC)	Closed (CC)	Open (OC)	Closed (CC)	Open (OC)	Closed (CC)	Open (OC)	Closed (CC)
StarVent2 (FM)	ΔP			3.20 ± 0.05				3.08 ± 0.03				2.91 ± 0.07	
	A_i			0.394 ± 0.005				0.556 ± 0.006				0.551 ± 0.005	
	A_e			0.310 ± 0.004				0.334 ± 0.003				0.363 ± 0.005	
	T%			44.9 ± 0.9				83.1 ± 1.2				89.2 ± 8	
Isleep 20 (OSA1)	ΔP	6.63 ± 0.10	8.31 ± 0.05	3.59 ± 0.06	7.04 ± 0.05	7.58 ± 0.07	10.22 ± 0.08	3.14 ± 0.03	8.37 ± 0.07	7.74 ± 0.08	8.92 ± 0.09	2.98 ± 0.04	6.81 ± 0.07
	A_i	0.791 ± 0.062	0.877 ± 0.006	0.570 ± 0.003	0.775 ± 0.003	1.014 ± 0.008	1.446 ± 0.004	0.767 ± 0.007	1.281 ± 0.006	1.105 ± 0.042	1.203 ± 0.009	0.800 ± 0.008	1.088 ± 0.007
	A_e	0.911 ± 0.081	0.776 ± 0.009	0.386 ± 0.003	0.637 ± 0.002	0.783 ± 0.007	1.357 ± 0.006	0.207 ± 0.003	1.113 ± 0.005	0.925 ± 0.009	1.258 ± 0.009	0.192 ± 0.004	1.018 ± 0.007
	T%	2.2 ± 0.9	97.7 ± 1.0	20.8 ± 0.8	75.8 ± 3.8	63.4 ± 0.7	96.7 ± 0.9	41.5 ± 1.3	89.0 ± 1.4	81.8 ± 0.5	93.7 ± 1.1	52.3 ± 0.9	90.7 ± 0.9
AirSense 10 (OSA2)	ΔP	3.88 ± 0.09	6.12 ± 0.07	2.74 ± 0.03	5.47 ± 0.07	5.14 ± 0.10	8.64 ± 0.10	2.4 ± 0.03	6.59 ± 0.04	5.95 ± 0.10	7.40 ± 0.08	2.53 ± 0.05	5.26 ± 0.07
	A_i	0.219 ± 0.004	0.685 ± 0.005	0.209 ± 0.007	0.500 ± 0.004	0.230 ± 0.006	0.970 ± 0.070	0.371 ± 0.005	0.743 ± 0.004	0.143 ± 0.009	0.805 ± 0.006	0.327 ± 0.006	0.576 ± 0.009
	A_e	0.213 ± 0.006	0.592 ± 0.006	0.451 ± 0.006	0.619 ± 0.005	0.291 ± 0.005	1.081 ± 0.006	0.226 ± 0.004	1.025 ± 0.005	0.353 ± 0.009	0.911 ± 0.004	0.203 ± 0.004	0.826 ± 0.006
	T%	88.9 ± 3.6	89.1 ±2.1	21.8 ± 0.7	97.2 ± 0.7	78.4 ± 1.8	89.5 ± 10.5	48.1 ± 1.4	99.0 ± 0.2	61.5 ± 3.6	95.0 ± 0.6	90.3 ± 1.6	92.1 ± 2.3
LUISA (V1)	ΔP	7.60 ± 0.2	9.36 ± 0.07	3.63 ± 0.04	7.26 ± 0.04	8.41 ± 0.07	9.72 ± 0.10	3.58 ± 0.04	8.20 ± 0.07	9.19 ± 0.07	10.18 ± 0.07	3.85 ± 0.04	7.87 ± 0.08
	A_i	0.513 ± 0.008	0.698 ± 0.010	0.285 ± 0.005	0.739 ± 0.006	0.668 ± 0.009	0.815 ± 0.006	0.484 ± 0.007	1.071 ± 0.026	0.671 ± 0.007	0.701 ± 0.010	0.567 ± 0.008	0.852 ± 0.007
	A_e	0.555 ± 0.027	1.309 ± 0.010	0.347 ± 0.005	0.646 ± 0.007	0.739 ± 0.005	1.740 ± 0.006	0.371 ± 0.004	1.101 ± 0.025	0.969 ± 0.006	1.931 ± 0.009	0.409 ± 0.005	1.078 ± 0.008
	T%	80.1 ± 1.8	97.6 ± 1.7	13.5 ± 22	86.3 ± 3.2	63.7 ± 0.6	95.1 ± 0.3	77.3 ± 1.4	96.2 ± 0.4	82.7 ± 0.9	92.3 ± 0.5	89.4 ± 0.8	85.3 ± 1.9
V60 ventilator (V2)	ΔP	5.72 ± 0.15	7.75 ± 0.13	2.93 ± 0.33	4.34 ± 0.09	5.92 ± 0.16	9.00 ± 0.13	3.93 ± 0.21	4.80 ± 0.08	5.99 ± 0.22	9.30 ± 0.06	4.27 ± 0.25	5.00 ± 0.05
	A_i	0.942 ± 0.011	0.630 ± 0.019	0.276 ± 0.016	0.421 ± 0.021	0.804 ± 0.013	0.765 ± 0.013	0.250 ± 0.040	0.639 ± 0.066	0.772 ± 0.017	0.695 ± 0.031	0.340 ± 0.037	0.736 ± 0.018
	A_e	0.624 ± 0.005	0.877 ± 0.012	0.107 ± 0.035	0.489 ± 0.009	0.687 ± 0.011	1.477 ± 0.015	0.329 ± 0.032	0.600 ± 0.012	0.776 ± 0.012	1.646 ± 0.009	0.420 ± 0.044	0.749 ± 0.008
	T%	61.7 ± 4.4	96.1 ± 2.2	60.9 ± 17.8	46.7 ± 0.9	90.9 ± 1.5	94.2 ± 0.4	69.5 ± 10.8	90.6 ± 2.0	89.1 ± 1.2	93.8 ± 0.5	71.8 ± 4.2	92.7 ± 0.8
Hamilton G5 (V3)	ΔP	7.08 ± 0.32		4.86 ± 0.08		8.46 ± 0.84		4.62 ± 1.19		9.71 ± 1.10		6.23 ± 0.54	
	A_i	0.233 ± 0.053		0.544 ± 0.022		0.257 ± 0.060		0.581 ± 0.123		0.303 ± 0.050		0.631 ± 0.165	
	A_e	1.086 ± 0.120		0.583 ± 0.040		1.224 ± 0.125		0.566 ± 0.186		1.456 ± 0.010		1.003 ± 0.109	
	T%	88.4 ± 13.4		55.5 ± 17.1		97.7 ± 1.8		59.2 ± 12.9		97.3 ± 2		99.6 ± 0.7	
Evita V800 (V4)	ΔP	6.23 ± 0.50				10.37 ± 0.57				12.05 ± 1.07			
	A_i	0.434 ± 0.061				0.301 ± 0.066				0.368 ± 0.470			
	A_e	0.201 ± 0.022				0.641 ± 0.056				0.807 ± 0.012			
	T%	44.4 ± 4.5				71.9 ± 5.1				77.8 ± 2.3			

Table S4: ΔP, A_i, A_e and T% results (mean ± standard deviation) for all performed tests at 10 cmH_2O

		Healthy				Post-surgery				ARDS			
		Mask		Helmet		Mask		Helmet		Mask		Helmet	
		Open (OC)	Closed (CC)	Open (OC)	Closed (CC)	Open (OC)	Closed (CC)	Open (OC)	Closed (CC)	Open (OC)	Closed (CC)	Open (OC)	Closed (CC)
**StarVent2 (FM)**	**ΔP**			3.37 ± 0.04				3.24 ± 0.05				3.11 ± 0.06	
	**A_i**			0.363 ± 0.003				0.532 ± 0.006				0.537 ± 0.008	
	**A_e**			0.254 ± 0.004				0.406 ± 0.005				0.426 ± 0.005	
	**T%**			71.1 ± 5.3				92.4 ± 4.1				90.8 ± 0.7	
**Isleep 20 (OSA1)**	**ΔP**	5.44 ± 0.09	8.02 ± 0.10	4.80 ± 0.04	6.5 ± 0.08	7.35 ± 0.05	9.90 ± 0.59	4.68 ± 0.06	8.04 ± 0.06	7.68 ± 0.06	8.85 ± 0.09	4.58 ± 0.06	6.53 ± 0.07
	**A_i**	0.102 ± 0.003	0.945 ± 0.015	0.754 ± 0.003	0.805 ± 0.003	0.995 ± 0.005	1.531 ± 0.035	0.721 ± 0.004	1.284 ± 0.006	1.104 ± 0.009	1.286 ± 0.006	0.747 ± 0.009	1.069 ± 0.006
	**A_e**	0.795 ± 0.006	0.761 ± 0.016	0.338 ± 0.003	0.614 ± 0.005	0.519 ± 0.010	1.241 ± 0.004	0.343 ± 0.005	1.022 ± 0.006	0.690 ± 0.012	1.172 ± 0.007	0.423 ± 0.007	0.949 ± 0.006
	**T%**	22.2 ± 1.6	98.0 ± 2.3	26.7 ± 8.1	91.1 ± 2.6	57.8 ± 1.5	98.6 ± 0.4	84.5 ± 2.1	99.0 ± 0.2	68.2 ± 1.2	94.5 ± 0.8	91.1 ± 1.7	92.8 ± 0.6
**AirSense 10 (OSA2)**	**ΔP**	5.26 ± 0.30	5.89 ± 0.08	3.69 ± 0.04	4.60 ± 0.07	5.31 ± 0.10	8.63 ± 0.10	4.58 ± 0.20	6.56 ± 0.04	5.83 ± 0.12	7.36 ± 0.60	5.32 ± 0.07	5.30 ± 0.04
	**A_i**	0.216 ± 0.050	0.683 ± 0.004	0.316 ± 0.005	0.514 ± 0.003	0.298 ± 0.005	1.076 ± 0.004	0.353 ± 0.005	0.809 ± 0.005	0.234 ± 0.008	0.891 ± 0.006	0.301 ± 0.007	0.624 ± 0.009
	**A_e**	0.283 ± 0.002	0.601 ± 0.006	0.274 ± 0.003	0.526 ± 0.004	0.264 ± 0.004	1.047 ± 0.005	0.550 ± 0.030	0.964 ± 0.004	0.344 ± 0.005	0.899 ± 0.006	0.522 ± 0.006	0.801 ± 0.006
	**T%**	52.3 ± 14	95.6 ± 1.8	53.8 ± 1.3	98.9 ± 1.5	27.9 ± 1.8	99.2 ± 0.4	49.2 ± 1.0	97.2 ± 0.2	64.1 ± 2.5	94.8 ± 2.7	37.1 ± 0.8	92.7 ± 0.5
**LUISA (V1)**	**ΔP**	5.85 ± 0.10	8.53 ± 0.08	4.71 ± 0.07	7.37 ± 0.04	8.3 ± 0.07	9.65 ± 0.10	4.81 ± 0.03	8.30 ± 0.09	9.035 ± 0.06	9.42 ± 0.01	5.51 ± 0.05	8.38 ± 0.10
	**A_i**	0.531 ± 0.005	0.730 ± 0.009	0.494 ± 0.007	0.745 ± 0.005	0.631 ± 0.006	0.781 ± 0.009	0.499 ± 0.006	1.060 ± 0.087	0.626 ± 0.009	1.086± 0.010	0.543 ± 0.007	1.136 ± 0.004
	**A_e**	0.357 ± 0.005	1.108 ± 0.011	0.326 ± 0.003	0.642 ± 0.006	0.610 ± 0.005	1.747 ± 0.030	0.503 ± 0.010	1.137 ± 0.019	0.844 ± 0.007	1.869 ± 0.009	0.598 ± 0.010	1.491 ± 0.010
	**T%**	64.1 ± 3.3	98.9 ± 0.8	25.3 ± 1.5	72.0 ± 4.3	62.4 ± 2.0	95.5 ± 0.5	57.8 ± 1.3	95.8 ± 0.3	72.9 ± 1.1	83.7 ± 27.2	80.8 ± 1.0	78.5 ± 4.7
**V60 ventilator (V2)**	**ΔP**	5.53 ± 0.20	6.51 ± 0.20	2.95 ± 0.56	3.78 ± 0.14	5.89 ± 0.17	8.87 ± 0.08	4.11 ± 0.46	4.65 ± 0.05	5.86 ± 0.18	9.30 ± 0.10	4.50 ± 0.31	5.11 ± 0.09
	**A_i**	0.538 ± 0.007	0.569 ± 0.040	0.297 ± 0.130	0.364 ± 0.015	0.821 ± 0.027	0.774 ± 0.015	0.218 ± 0.057	0.491 ± 0.030	0.931 ± 0.010	0.706 ± 0.012	0.332 ± 0.044	0.527 ± 0.020
	**A_e**	0.316 ± 0.006	0.668 ± 0.030	0.158 ± 0.126	0.332 ± 0.016	0.685 ± 0.028	1.424 ± 0.008	0.369 ± 0.034	0.648 ± 0.010	0.612 ± 0.035	1.602 ± 0.009	0.404 ± 0.042	0.792 ± 0.010
	**T%**	86.5 ± 2	78.7 ± 7.1	47.8 ± 16.8	47.3 ± 3.5	95.8 ± 2.5	96.5 ± 0.5	75.5 ± 4.1	92.5 ± 1.7	71.3 ± 3.9	90.8 ± 0.7	67.7 ± 4.3	95.8 ± 2.4
**Hamilton G5 (V3)**	**ΔP**	7.48 ± 1.20				8.22 ± 0.35				9.34 ± 0.11			
	**A_i**	0.312 ± 0.131				0.312 ± 0.040				0.289 ± 0.027			
	**A_e**	1.052 ± 0.270				0.947 ± 0.084				1.180 ± 0.0.24			
	**T%**	55.6 ±14.6				90.3 ± 4.5				84.1 ± 2.0			
**Evita V800 (V4)**	**ΔP**					10.24 ± 0.44				11.48 ± 0.61			
	**A_i**					0.353 ± 0.039				0.404 ± 0.025			
	**A_e**					0.685 ± 0.061				0.868 ± 0.025			
	**T%**					66.7 ± 3.6				66.7 ± 2.7			

Table S5: Factorial ANOVA table for the ΔP parameter

**Source**	**Sum Sq**	**D.F.**	**Mean Sq**	**F**	**Prob>F**
Set CPAP level	3.62	2	1.81	5.53	4.1E-03
Configuration	1222.26	1	1222.26	3737.86	0.0E+00
Simulated clinical condition	144.66	2	72.33	221.19	7.9E-80
Interface	1590.11	1	1590.11	4862.80	0.0E+00
Device	759.50	3	253.17	774.23	1.7E-256
Set CPAP level - Configuration	18.21	2	9.11	27.85	1.7E-12
Set CPAP level - Simulated clinical condition	28.41	4	7.10	21.72	3.5E-17
Set CPAP level - Interface	28.17	2	14.08	43.07	1.2E-18
Set CPAP level - Device	13.74	6	2.29	7.00	2.6E-07
Configuration - Simulated clinical condition	54.72	2	27.36	83.67	3.1E-34
Configuration - Interface	4.34	1	4.34	13.28	2.8E-04
Configuration - Device	17.49	3	5.83	17.83	2.9E-11
Simulated clinical condition - Interface	35.51	2	17.76	54.30	4.5E-23
Simulated clinical condition - Device	29.62	6	4.94	15.10	1.4E-16
Interface - Device	40.23	3	13.41	41.01	6.0E-25
Error	316.20	967	0.33		
Total	4306.79	1007			

Table S6: Factorial ANOVA table for the A_i parameter

**Source**	**Sum Sq**	**D.F.**	**Mean Sq**	**F**	**Prob>F**
Set CPAP level	0.27	2	0.13	11.76	9.0E-06
Configuration	22.30	1	22.30	1976.49	5.7E-236
Simulated clinical condition	8.71	2	4.36	386.24	5.1E-124
Interface	3.82	1	3.82	338.66	4.5E-65
Device	30.45	3	10.15	899.88	2.9E-279
Set CPAP level - Configuration	0.02	2	0.01	0.76	4.7E-01
Set CPAP level - Simulated clinical condition	0.09	4	0.02	1.93	1.0E-01
Set CPAP level - Interface	0.05	2	0.03	2.35	9.6E-02
Set CPAP level - Device	0.60	6	0.10	8.86	2.0E-09
Configuration - Simulated clinical condition	1.50	2	0.75	66.35	1.0E-27
Configuration - Interface	0.32	1	0.32	28.02	1.5E-07
Configuration - Device	4.04	3	1.35	119.51	7.8E-66
Simulated clinical condition - Interface	0.10	2	0.05	4.27	1.4E-02
Simulated clinical condition - Device	4.41	6	0.74	65.20	4.5E-68
Interface - Device	3.58	3	1.20	105.93	2.7E-59
Error	10.91	967	0.01		
Total	91.17	1007			

Table S7: Factorial ANOVA table for the A_e parameter

Source	Sum Sq	D.F.	Mean Sq	F	Prob>F
Set CPAP level	2.75	2	1.38	114.52	2.3E-45
Configuration	73.57	1	73.57	6124.15	0.0E+00
Simulated clinical condition	21.90	2	10.95	911.45	3.2E-223
Interface	26.99	1	26.99	2246.45	2.1E-254
Device	20.28	3	6.76	562.79	1.5E-211
Set CPAP level - Configuration	0.36	2	0.18	15.13	3.4E-07
Set CPAP level - Simulated clinical condition	0.06	4	0.02	1.31	2.6E-01
Set CPAP level - Interface	0.80	2	0.40	33.09	1.3E-14
Set CPAP level - Device	0.39	6	0.06	5.36	1.9E-05
Configuration - Simulated clinical condition	5.96	2	2.98	247.95	1.2E-87
Configuration - Interface	1.94	1	1.94	161.76	2.2E-34
Configuration - Device	2.28	3	0.76	63.34	2.2E-37
Simulated clinical condition - Interface	2.07	2	1.04	86.27	3.3E-35
Simulated clinical condition - Device	2.39	6	0.40	33.12	2.0E-36
Interface - Device	8.14	3	2.71	225.83	5.1E-111
Error	11.62	967	0.01		
Total	181.49	1007			

Table S8: Factorial ANOVA table for the T% parameter

Source	Sum Sq	D.F.	Mean Sq	F	Prob>F
Set CPAP level	653.10	2	326.50	1.84	1.6E-01
Configuration	203006.20	1	203006.20	1142.14	6.2E-166
Simulated clinical condition	85802.70	2	42901.30	241.37	9.3E-86
Interface	35504.60	1	35504.60	199.75	2.3E-41
Device	8270.20	3	2756.70	15.51	7.4E-10
Set CPAP level - Configuration	3257.40	2	1628.70	9.16	1.1E-04
Set CPAP level - Simulated clinical condition	3211.20	4	802.80	4.52	1.3E-03
Set CPAP level - Interface	1088.30	2	544.20	3.06	4.7E-02
Set CPAP level - Device	19865.70	6	3310.90	18.63	1.4E-20
Configuration - Simulated clinical condition	12639.80	2	6319.90	35.56	1.3E-15
Configuration - Interface	2625.40	1	2625.40	14.77	1.3E-04
Configuration - Device	20058.40	3	6686.10	37.62	5.4E-23
Simulated clinical condition - Interface	15168.00	2	7584.00	42.67	1.7E-18
Simulated clinical condition - Device	34895.80	6	5816.00	32.72	5.3E-36
Interface - Device	13350.30	3	4450.10	25.04	1.3E-15
Error	171876.00	967	177.70		
Total	631273.20	1007			
